# In Utero Diagnoses of Strikingly Similar Presentations of Complete Atrioventricular Septal Defects in a Pair of Dizygotic Twins Concordant for Trisomy 21

**DOI:** 10.1155/2018/6215675

**Published:** 2018-10-17

**Authors:** Diamond Ling, Jonathan G Dayan

**Affiliations:** ^1^Department of Pediatrics, UC-Davis Children's Hospital, Sacramento, California, USA; ^2^Division of Cardiology, UC-Davis Children's Hospital, Sacramento, California, USA

## Abstract

Trisomy 21, or Down syndrome (DS), is a genetic disorder affecting approximately 1 in 500–750 live births. The prevalence of DS has increased over the past two decades, correlating with a rise in the proportion of pregnancies complicated by advanced maternal age. There is also a correlation between advanced maternal age and dizygotic twinning rates. There is an increased risk of at least one twin being affected in dizygotic pregnancies compared to singletons. However, despite this greater relative risk, reports of concordance of DS in both dizygotic twins are very rare. Congenital heart disease (CHD) occurs in roughly 40% of individuals with DS, but there can be considerable phenotypic variation. The most common, atrioventricular septal defect accounts for only 40% of CHD seen in DS. There is also a higher incidence of CHD in twins, but also with a low incidence of concordance. There have been only five reported cases of concordant DS in dizygotic twins with confirmed chromosomal analyses; none of which describe concordant congenital heart disease. Here, we describe an unusual case of dizygotic twins of differing genders concordant for both Down syndrome and congenital heart disease of a strikingly similar presentation.

## 1. Introduction

Trisomy 21, or Down syndrome (DS), is a genetic disorder affecting approximately 1 in 500–750 live births [1, 2]. The majority of cases occur by whole-chromosome trisomies caused by nondisjunction events, while other types include Robertsonian translocations, segmental duplication, and single gene duplications [1]. The prevalence of DS has increased over the past two decades, correlating with a rise in the proportion of pregnancies complicated by advanced maternal age [2, 3]. A correlation has also been made between advanced maternal age and dizygotic twinning rates [4]. Interestingly, there is a lower risk of DS in monozygotic twin gestations relative to singleton pregnancies [2, 5], but an increased risk (∼1/3-fold) of at least one twin being affected in dizygotic pregnancies [2]. Despite this greater relative risk, reports of concordance of DS in both dizygotic twins are very rare [2, 6].

The incidence of congenital heart defects (CHD) is approximately 8 in every 1,000 live births [7]. In contrast, CHD occurs in roughly 40% of individuals with DS [1]. There can be considerable phenotypic variation; the most common presentation, atrioventricular septal defect (AVSD), accounts for approximately 40% of CHD in DS patients compared to approximately 3–5% of CHD in non-DS patients [1, 8, 9]. There is also a higher incidence of CHD in twins, with a 6–10 fold increase in prevalence of CHD among twin gestations compared to singletons [10]. The prevalence of CHD concordance in twin gestations with presence of CHD is ∼9.5% in monozygotic twins and 4.5–13.5% in dizygotic twins [11, 12].

There have been only five reported cases of concordant DS in dizygotic twins with confirmed chromosomal analyses [2, 6, 13]. There have also been a small number of historical reports of presumed dizygotic twins concordant for DS based on clinical examination alone [14–17]. CHD is not described in these cases. Here, we describe an unusual case of dizygotic twins of differing genders concordant for both DS and CHD of a strikingly similar presentation.

## 2. Case

A 37-year-old, gravida 3, para 2 woman was referred for fetal echocardiography due to prenatal ultrasounds that showed a dichorionic/diamniotic twin gestation with the following anomalies: Twin A (female) had a thickened nuchal fold, absent nasal bone, small stomach, and complex CHD consisting of a ventricular septal defect (VSD), atrial septal defect (ASD), pericardial effusion, deviated cardiac axis, and possible AVSD; Twin B (male) had all of the above noted as well as short long bones. These findings were concerning for DS in both twins. Previous pregnancies were delivered via normal spontaneous vaginal delivery and the children did not have genetic or congenital conditions. Amniocentesis was declined during the current pregnancy due to maternal concern for associated risks.

**Figure 1:**
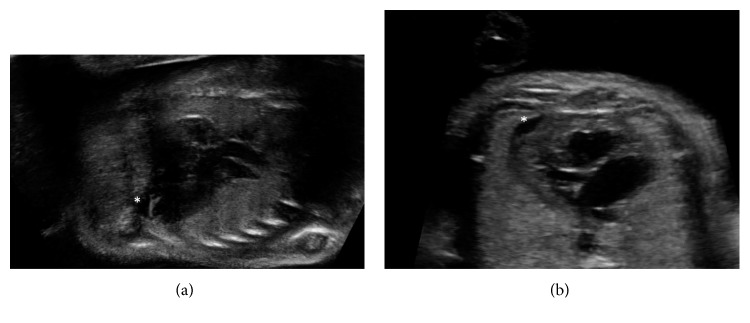
Fetal pericardial effusions. Fetal Twin A (a) and fetal Twin B (b) showing similar small apical pericardial effusions (∗).

**Figure 2:**
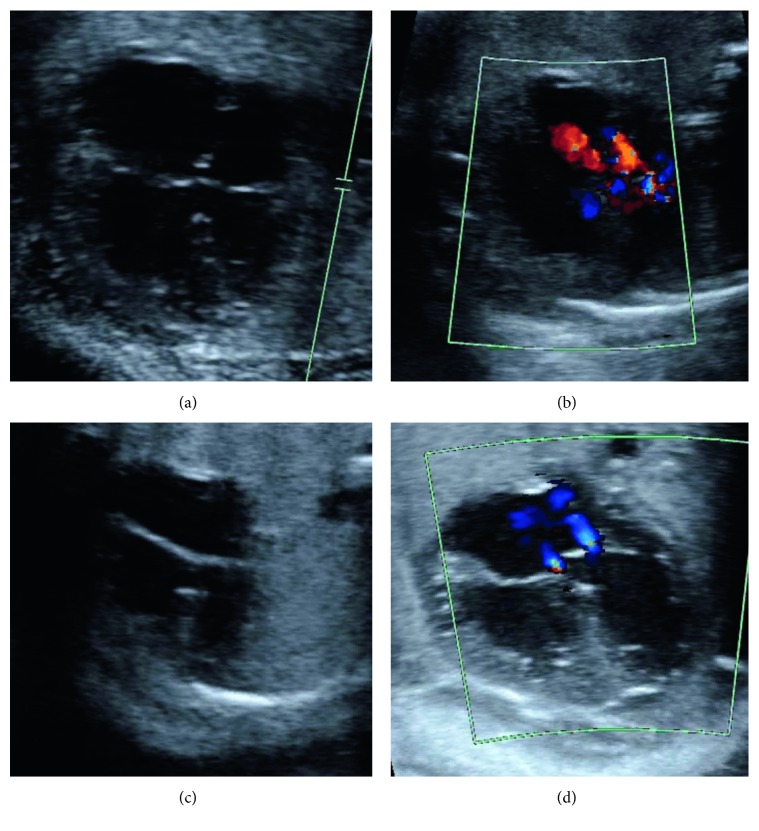
Fetal four-chamber cardiac images. (a) Twin A 2D imaging showing a ventricular septal defect, primum atrial septal defect, and secundum atrial septal defect. (b) Twin A color Doppler showing left and central atrioventricular valve regurgitation (red flashes moving toward the transducer). (c) Twin B 2D imaging showing a ventricular septal defect, primum atrial septal defect, and secundum atrial septal defect (drop out artifact between these defects gives the incorrect appearance of a large single defect). (d) Twin B color Doppler showing left and central atrioventricular valve regurgitation (blue flashes moving away from the transducer).

Fetal echocardiography was performed initially at 27 weeks and 2 days gestation, showing each twin had a complete, balanced AVSD of Rastelli type A consisting of a moderate-sided inlet VSD, a small primum ASD, a probable small secundum ASD, and a single atrioventricular valve with trivial left-sided and mild central atrioventricular valve regurgitation and a small predominantly apical pericardial effusion (Figures 1 and 2(a)–2(d)). Biventricular size and qualitative systolic function were normal in both twins, as was conotruncal anatomy and aortic and ductal arch anatomy.

Follow-up obstetric ultrasound at 30 weeks gestation was notable for oligohydramnios, mild ascites, and severe growth restriction in Twin A and polyhydramnios in Twin B. Estimated fetal weight for Twin A was 18th percentile and for Twin B was 50th percentile. The mother received two treatments of betamethasone at that time. The twins were closely followed and the ascites in Twin A was noted to improve over time, but they were ultimately delivered at 33 weeks gestation via emergent cesarean section due to nonreassuring heart tracings in Twin A.

**Figure 3:**
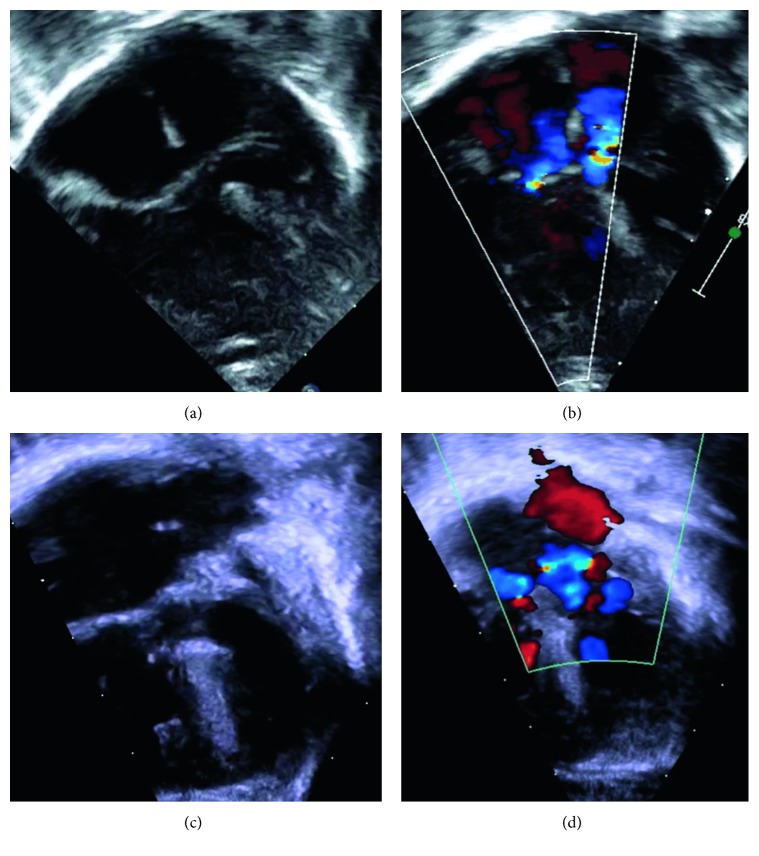
Postnatal four-chamber cardiac images. (a) Twin A 2D imaging showing a ventricular septal defect, primum atrial septal defect, and secundum atrial septal defect. (b) Twin A color Doppler showing left, central, and right atrioventricular valve regurgitation (blue flashes moving away from the transducer. (c) Twin B 2D imaging showing a ventricular septal defect, primum atrial septal defect, and secundum atrial septal defect. (d) Twin B color Doppler showing left and right atrioventricular valve regurgitation (blue flashes moving away from the transducer).

Birth measurement of Twin A was at the ∼5th percentile for length (39 cm), weight (1410 g), and occipitofrontal head circumference (27.5 cm), based on the Olsen Premature Girls Chart (which does not account for DS). Apgar scores at 1 and 5 min were 8 and 9, respectively. The patient was subsequently admitted to the neonatal intensive care unit (NICU) for low birth weight and mild respiratory distress requiring supplemental oxygen; she was noted to be 500 g smaller than her twin at birth. Notable dysmorphic features included transverse palmar crease on her left hand, tongue thrusting, a flat nasal bridge, and upslanting palpebral fissures. Her exam was also notable for suspected choanal atresia or stenosis of her right nare. Chromosomal analysis confirmed the diagnosis of DS with 47XX + 21 karyotype. Transthoracic echocardiography on day of life one confirmed the diagnosis of Rastelli type A complete AVSD with moderate atrial and ventricular septal defects, a balanced common atrioventricular valve with mild right and trace left atrioventricular valve regurgitation, and a patent foramen ovale (Figures 3(a) and 3(b)). Heart size was normal with low normal qualitative right ventricular systolic function and normal left ventricular size and systolic function. There was no evidence of left ventricular outflow tract obstruction, conotruncal anatomy was normal, the aortic arch appeared unobstructed, and there was a small patent ductus arteriosus with predominantly left to right shunting.

Birth measurement of Twin B was at the ∼20th percentile for length (41.5 cm), ∼42nd percentile for weight (1905 g), and ∼55th percentile of occipitofrontal head circumference (31 cm), based on the Olsen Premature Boys Chart (which does not account for DS). Apgar scores at 1 and 5 min were 7 and 9, respectively. The patient was subsequently admitted to the NICU for persistent respiratory distress requiring supplemental oxygen. Notable dysmorphic features included transverse palmar crease, macroglossia, a flat nasal bridge, low set ears, increased skin over back of neck, a short neck, and upslanting palpebral fissures. Chromosomal analysis confirmed diagnosis of DS with 47XY + 21 karyotype. Transthoracic echocardiogram on day of life one confirmed the diagnosis of Rastelli type A complete AVSD with a large atrial septal defect and a moderate ventricular septal defect, a balanced common atrioventricular valve with mild right and trace left atrioventricular valve regurgitation, and a patent foramen ovale (Figures 3(c) and 3(d)). Heart size was normal with low normal qualitative right ventricular systolic function and normal left ventricular size and systolic function. There was no evidence of left ventricular outflow tract obstruction, conotruncal anatomy was normal, the aortic arch appeared unobstructed, and there was a small patent ductus arteriosus with predominantly left to right shunting.

On day of life 25, diuretic therapy with furosemide was initiated in both twins for the management of persistent respiratory distress, consistent with pulmonary overcirculation, a common complication in AVSD. Repeat echocardiography at one month of age showed only modest changes—both twins had mild right atrial dilation, mild to moderate right ventricular dilation with moderate hypertrophy and good qualitative systolic function, normal left ventricular size, and systolic function, mild central and right atrioventricular valve regurgitation, and no evidence of a persistent PDA. The patients remained stable during their hospital course and were discharged after 41 days, and continued furosemide therapy at home.

Despite the similarities in these twins' CHD, they had divergent clinical courses. Twin B followed a typical course for AVSD and underwent complete surgical repair at seven months of age with relatively smooth postoperative course. Twin A, however, passed away from complication of presumed necrotizing enterocolitis with bowel perforation following admission for repair of her choanal atresia at three months of age.

## 3. Discussion

One major risk factor for DS is advanced maternal age at conception [18]. Over the past two decades, the total prevalence of DS during pregnancy has progressively increased over time, recently estimated to be ∼2 per 1,000 singletons and ∼1.5 per 1,000 multiples [2, 3]. Though prevalence of DS among twin gestations is lower compared to singletons, there is a higher relative risk of diagnosing at least one twin in dizygotic twin pregnancies with DS compared to singleton pregnancies and monozygotic twin pregnancies [2].

The prevalence of CHD is higher in twin gestations and in individuals with DS [1, 10]. Despite relatively higher prevalence of CHD in twin gestations compared to singleton pregnancies, concordance for CHD is relatively rare [11, 12]. Concordance for DS appears to be even less common among dizygotic twin gestations with only a few cases reported with confirmatory laboratory testing [2,6,13–17]. It can be inferred, therefore, that cases of dizygotic twins concordant for both DS and CHD are extremely rare.

There has been one case report of monozygotic twins with concordance for DS and CHD [19]. To the best of our knowledge, there have been no previously reported cases of dizygotic twins concordant for both CHD and DS. Recent literature suggests that presence of key genetic variants found on chromosome 21 significantly increases susceptibility to development of CHD, and even AVSD in particular, in DS [9, 20]. These variants include single gene polymorphisms, single gene defects, copy number variation, and microRNA overexpression, but no single variant alone is associated with development of an AVSD [9, 20]. Thus, it is also incredibly remarkable to note the highly similar presentations of AVSD in our reported dizygotic twins given this diversity of genotype and phenotype.

It is also interesting that despite their clinical similarities, these twins had very divergent courses that are beyond the scope of this paper.
